# Early visual intervention, visual function analysis, and grating visual acuity outcomes in children with congenital Zika syndrome

**DOI:** 10.5935/0004-2749.2022-0375

**Published:** 2024-09-16

**Authors:** Marcia Beatriz Tartarella, Ana Paula Braga, Jean Hipolito M. Borges, Rosana S.A. S. Furtado, Natalia Gomes Diogo, Islane M. C. Verçosa, Eduarda Tartarella-Nascimento

**Affiliations:** 1 CAVIVER Eye Clinic, Centro de Aperfeiçoamento Visual Ver a Esperança Renascer, Fortaleza, CE, Brazil; 2 Centro Integrado de Oftalmologia Tartarella, São Paulo, SP, Brazil

**Keywords:** Zika virus infection/congenital, Low vision, Vision disorders, Atrophy, Microcephaly, Visual acuity, Child

## Abstract

**Purpose:**

This study aimed to assess grating visual acuity and functional vision in children with congenital Zika syndrome.

**Methods:**

Initial and final grating visual acuity was measured using Teller acuity cards. Cerebral vision impairment standardized tests were used to assess functional vision. Patients were referred to the early visual intervention program for visually disabled children. Neuroimaging was performed.

**Results:**

In this study, 10 children were included with an age range of 1-37 months. Eight patients presented with macular atrophic scars. Neuroimaging revealed microcephaly and cerebral abnormalities in all patients. Low vision and cerebral vision impairment characteristics were observed in all children. The final grating visual acuity in this group varied from 3.00 to 0.81 logMAR.

**Conclusions:**

The grating visual acuity test revealed low vision in all children with congenital Zika syndrome. Functional vision evaluation revealed cerebral vision impairment characteristics in all patients, who were referred to the early visual intervention program. Visual acuity improved in six children.

## INTRODUCTION

Congenital Zika syndrome (CZS) results from maternal exposure to the Zika virus during pregnancy^(1)^. Clinical characteristics of CZS include microcephaly, brain malformation, arthrogryposis, and atrophic retinal lesions^(1^,^2)^. Macular atrophic lesions and optic nerve hypoplasia or atrophy were observed in newborns with CZS^(3)^. Studies have revealed that ocular findings and neurological anomalies associated with CZS cause visual impairment^(4)^.

The objectives of this case series were to assess grating visual acuity (VA), analyze functional vision, and describe the early visual intervention program implemented for children with CZS.

## METHODS

A descriptive analysis of a case series was conducted at the CAVIVER Institute in the city of Fortaleza, CE, Brazil. Patients with microcephaly due to the Zika virus were evaluated by a team comprising ophthalmologists, orthoptists, physical therapists, neurologists, geneticists, orthopedists, and pediatricians. The selection criteria included patients with microcephaly analyzed through brain neuroimaging; those with negative serum testing for toxoplasmosis, rubella, syphilis, and herpes simplex virus (STORCHS); and those with normal genetic study.

All patients underwent comprehensive ophthalmic evaluation. Strabismus was assessed using the cover test. Monocular and binocular best-corrected grating VA was obtained using the Teller acuity card test (Teller Acuity Cards II^®^) at 38 cm and age-matched according to a standardized graphic of normative values^(5)^.

**Figure 1:**
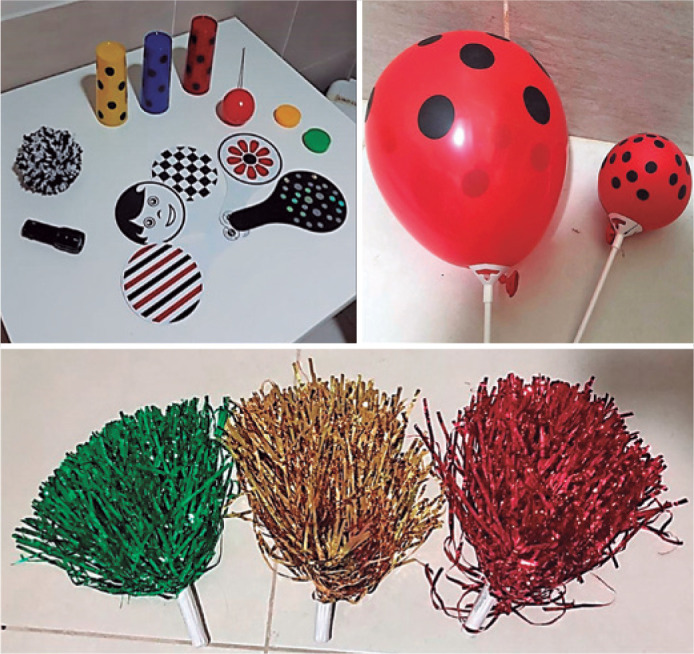
Functional vision evaluation kit.

Functional vision was assessed according to cerebral visual impairment (CVI) evaluation^(6)^. Seven characteristics of CVI behavior were analyzed: 1) visual latency; 2) color preference; 3) eye tracking of moving objects; 4) visual field preferences; 5) photophobia; 6) light gazing and staring at lights; and 7) visual response to near and distant objects (at 40 and 100 cm, respectively). Customized materials to evaluate the CVI characteristics were as follows: red with black dots, green and yellow balloons, and laminated pompons (Figure 1).

The early visual intervention program was administered by orthoptists and physical and occupational therapists, and the sessions were scheduled twice a month. This study was approved by the Ethics Committee of Albert Sabin Pediatric Hospital and adhered to the principles of the Declaration of Helsinki.

## RESULTS

In this study, 10 children, with an age range of 1-37 months (mean age=20 ± 11.62 months; median age=23 months), were included. None were born prematurely. Six patients were female. The mean follow-up duration was 20 months. Atrophic macular colobomatous-like lesions were detected in eight patients, and optic nerve hypoplasia was detected in four patients. Nine patients presented with strabismus (exodeviation: 6 cases; esodeviation: 3 cases), and eight patients had nystagmus. The refractive status ranged from +3.50 to -14.00 diopters of spherical equivalent. Glasses were prescribed to nine patients.

The final bilateral grating VA varied from 3.00 to 0.81 logMAR. The best-corrected bilateral initial and final grating VA values are presented in table 1. The monocular grating VA and deficits related to age are shown in the graphs for each patient. All patients presented with at least five CVI characteristics. Visual latency was observed in seven patients.

**Table 1:** Ocular characteristics of patients with congenital Zika syndrome

Patients	Initial age	Final age	Refraction RE/LE		Initial Va Logmar	Final Va Logmar	Initial Va Snellen	Final Va Snellen	Macular atrophy	Optic nerve hypoplasia	Exo deviation	Eso deviation	Nystagmus
1	5	38	-14.00	-10.00	0.65	0.95	20/89	20/180	0	1	0	1	0
2	28	35	-2.75-2.50 (20^o^)	-1.25 (180^o^)	2.28	2.13	20/3800	20/2700	1	1	1	0	1
3	17	44	+ 2.50	+1.50	2.00	1.52	20/2000	20/670	1	0	1	0	1
4	12	39	-4.50-2.75 (180^o^)	+3.50	1.52	1.25	20/670	20/360	1	1	0	1	1
5	25	46	-1.50-1.00 (180^o^)	-1.50-1.00 (180^o^)	0.67	1.11	20/94	20/260	1	0	0	0	0
6	21	34	-1.00-1.75 (180^o^)	-0.75-3.00 (180^o^)	1.52	1.25	20/670	20/360	0	1	1	0	1
7	29	33	+6.00-3.50 (150^o^)	+6.00-3.50 (180^o^)	1.25	0.81	20/360	20/130	1	0	1	0	1
8	30	44	-3.00	-1.00	2.24	1.52	20/3500	20/670	1	0	1	0	1
9	1	46	+1.00-3.75 (160^o^)	+2.50-2.00 (165^o^)	1.52	1.52	20/670	20/670	1	1	0	1	1
10	37	44	+2.00-2.50 (170^o^)	+3.00-3.00 (10^o^)	3.00	3.00	LP	LP	1	0	1	0	1
**Total of cases**									**8**	**4**	**6**	**3**	**8**

RE= right eye; LE= left eye. Occurrence: 0: none= 1: yes. LP= light perception; VA= bilateral visual acuity.

Age (in months).

**Figure 2:**
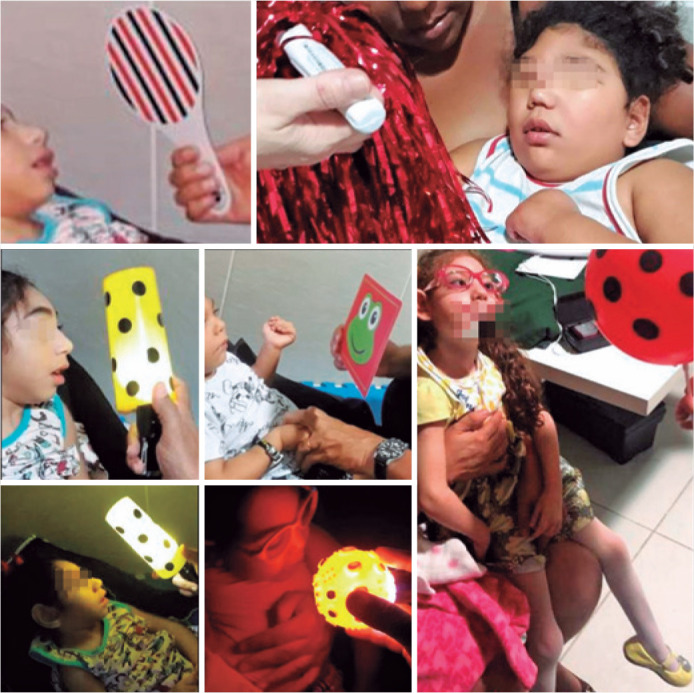
Early visual intervention.

Each child had unique needs, and early visual intervention protocols were individually crafted according to visual function and motor ability. The visits were scheduled twice a month; however, the intervals between sessions varied according to each child’s health condition. The sessions included visual activities using different contrast cards. Colorful objects and age-appropriate toys were used. Transilluminated gas balloons placed 20-40 cm away from the child were used as moving targets to induce eye tracking. Visual activities were performed in a room with natural light and repeated in a dark room with self-illuminated toys (Figure 2).

## DISCUSSION

Children with CZS may present with microcephaly, brain atrophy, motor disabilities, macular atrophic lesions, and optic nerve hypoplasia^(7)^.

The initial and final grating VA values were below the normal range in all patients in this study. Poor cervical control and milestone delay made it difficult to perform both VA tests and visual function evaluation. Visual impairment in patients with CZS may be because of multiple anatomical and sensory mechanisms of amblyopia, including ocular anatomical abnormalities, strabismus, nystagmus, refractive errors, and severe cerebral atrophy^(8)^. The VA improvement in six patients might have resulted from aging development (Table 1). Considering the age range of the patients, neuroplasticity of the visual system may have optimized the results of early visual intervention. The early visual intervention program was implemented to increase the use of the children’s potential residual vision in daily life activities. Two patients (patients 1 and 5) presented with decreased final VA. This might be correlated with a lack of attention because no changes in ocular or neurological status were observed; however, this is difficult to prove.

A study analyzed expected visual milestones based on age and contrast sensitivity tests, and according to the results, 26% of children with CZS had visual impairment^(9)^.

The characteristics of functional vision in patients with CZS were similar to those in other patients with CVI^(10)^. The CVI characteristics observed in this group, particularly visual latency, which was the most prevalent characteristic in this sample, represented a challenge for family members and the visual rehabilitation team. In CVI, the type, form, and duration of visual stimulus presentation and its response time must be individually adapted to maximize the use of residual vision.

Visual intervention techniques, protocols, and guidelines must be adapted for severe motor disabilities and cognitive and neurological impairment. These children required special devices, such as wheelchairs, parapodium, special postural helper chairs, and cervical vests. Multidisciplinary assessment is important. An integrated team approach that includes family, caretakers, doctors, and therapists was essential for caring for patients with multiple disabilities caused by CZS. Systemic clinical conditions, seizures, or challenges regarding transportation prevented the patients from attending all appointments. Consultations by telemedicine could be of great value to patients with multiple disabilities. Families were encouraged to replicate the visual activities at home. These strategies can reduce the need for several visits to the clinic.

This case series comprised a small sample size and had some limitations, such as the lack of contrast sensitivity testing and a control group.

In this case series, six children improved their final VA. Visual latency was a frequent CVI characteristic in this group of patients. The early visual intervention program was helpful in enhancing the possibilities to make better use of residual vision and may be a step toward improving the quality of life of children with CZS.
